# Case Report: Progressive limb necrosis as a portal to multifocal osteomyelitis in pediatric septic shock: a tissue-oriented management paradigm

**DOI:** 10.3389/fped.2026.1762616

**Published:** 2026-05-08

**Authors:** Cheng Yang, Wansha Zhou, Ximeng Huang, Jiawei Li

**Affiliations:** 1 Department of Pediatric Intensive Care Unit Nursing, WCSUH-Tianfu·Sichuan Provincial Children's Hospital, Meishan, China; 2 Department of Pediatric Intensive Care Unit Nursing, West China Second University Hospital, Sichuan University, Chengdu, China; 3 Key Laboratory of Birth Defects and Related Diseases of Women and Children (Sichuan University), Ministry of Education, Chengdu, China; 4 Department of Pediatric Outpatient Nursing, West China Second University Hospital, Sichuan University, Chengdu, China

**Keywords:** limb necrosis, multidisciplinary collaboration, osteomyelitis, septic shock, tissue viability, wound infection

## Abstract

**Background:**

Limb necrosis in pediatric septic shock may serve as a persistent source of deep infection, but its role in hematogenous dissemination is underrecognized.

**Case presentation:**

A 12-year-old boy with septic shock developed progressive left foot necrosis. This was followed by septic arthritis, multifocal osteomyelitis with extensive osteolytic destruction and pathological fracture of the femur, recurrent pneumothorax, and deep vein thrombosis, complicated by multidrug-resistant infections.

**Management strategies:**

Source control was achieved via ultrasound-guided drainage of septic arthritis, pathogen-directed antibiotics, and multidisciplinary conservative management prioritizing medical treatment over high-risk surgery.

**Results:**

Clinical stabilization occurred after more than 90 days of intensive care before transfer to a general ward.

**Conclusion:**

This case illustrates that limb necrosis in septic shock may precede disseminated osteomyelitis. Early bedside ultrasound and multidisciplinary source control are essential when deep infection is suspected.

## Introduction

Septic shock remains a life-threatening condition in the pediatric intensive care unit, with complex pathophysiology involving multiple organ dysfunction (1, 2). Limb necrosis in this setting is typically considered a consequence of microcirculatory failure (3). However, such necrotic foci may become persistent sources of deep infection, potentially seeding hematogenous dissemination to bones and joints (4). This report describes a pediatric septic shock case that progressed from localized limb necrosis to multifocal osteomyelitis with pathological fracture, highlighting the importance of early tissue viability assessment and multidisciplinary management.

## Case presentation

### Admission and initial resuscitation

A 12-year-and-10-month-old boy was admitted on July 28, 2024, with one week of fever, generalized myalgia, and three days of abdominal pain and shortness of breath. On admission, he appeared critically ill: temperature 38.1℃, heart rate 154 beats/min, respiratory rate 60 breaths/min, blood pressure 122/65 mmHg, and oxygen saturation 86% on facemask oxygen. Physical examination revealed mottled skin, cold extremities, capillary refill time of 4–5 s, and tachypnea with intercostal retractions. CT from an external hospital showed bilateral pulmonary infiltrates and pleural effusion. Laboratory tests showed elevated C-reactive protein (188.52 mg/L), hyperlactatemia (8.5 mmol/L), abnormal liver and renal function, and coagulopathy. Preliminary diagnoses included septic shock, severe pneumonia, respiratory failure, and multiple organ dysfunction syndrome.

The patient was transferred to the PICU, intubated, and mechanically ventilated. Fluid resuscitation was initiated, and norepinephrine combined with epinephrine was administered. Continuous renal replacement therapy was started on the following day.

### Disease progression

#### Limb necrosis

**Figure 1:**
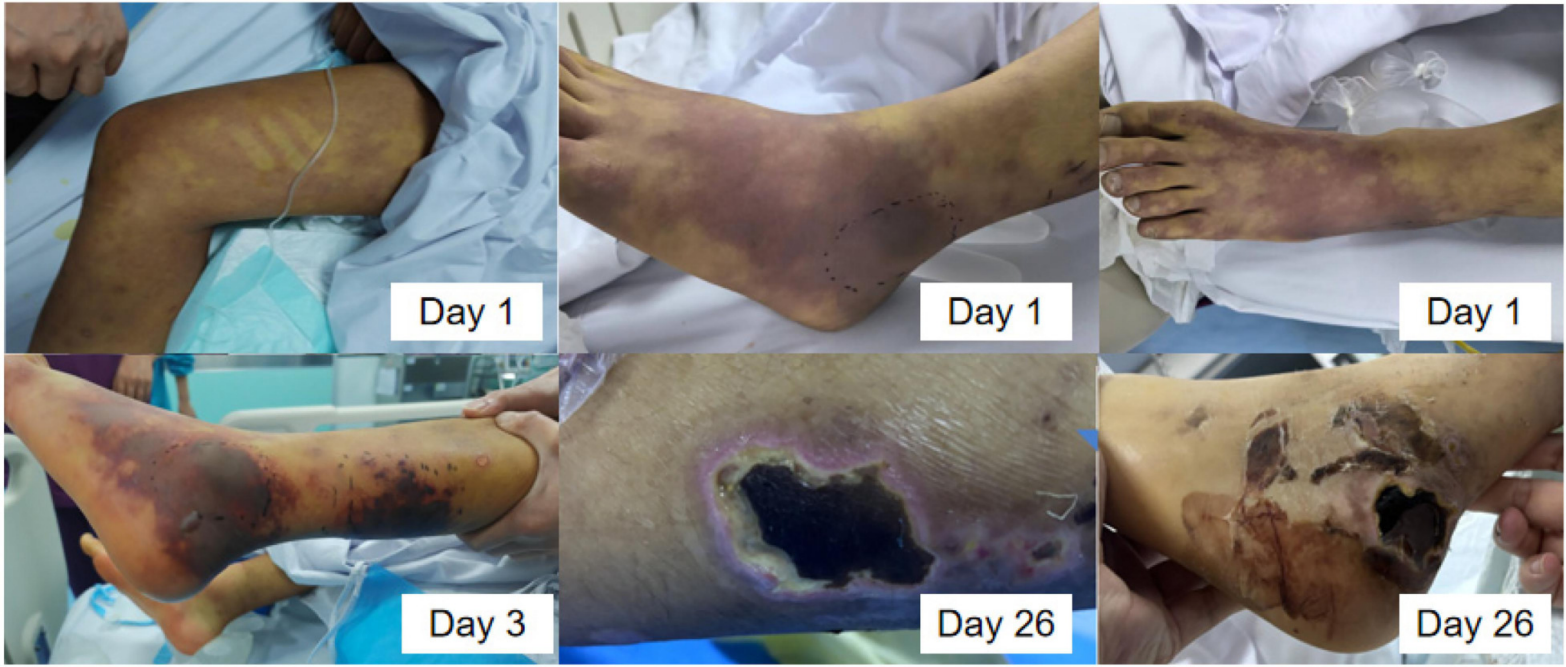
Necrosis of the skin on the left foot and calf.

On day 3, the child developed scattered ecchymoses on both feet, followed by progressive necrosis and suppuration of the left foot and calf (Figure 1).

#### Deep infection and hematogenous dissemination

**Figure 2:**
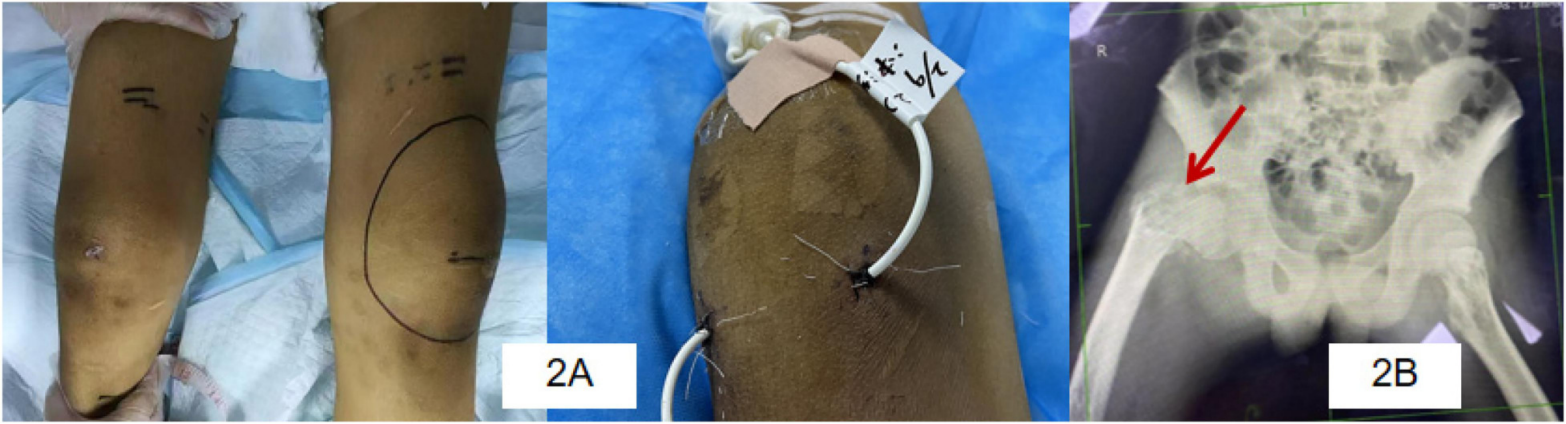
**(A)** knee cellulitis; **(B)** Osteolytic destruction of the right femur.

On day 29, left knee swelling was noted. Bedside ultrasound revealed a large effusion; 60 ml of purulent fluid was aspirated under ultrasound guidance, followed by an additional 52 ml on day 28, confirming septic arthritis/cellulitis (Figure 2A). Chest CT on day 54 revealed multiple osteolytic lesions, sequestrum formation, and a pathological fracture of the right femur (Figure 2B), establishing the diagnosis of hematogenously disseminated multifocal osteomyelitis. Metagenomic sequencing from blood and sputum identified methicillin-resistant Staphylococcus aureus (MRSA) as the likely progenitor (5). Co-infection with non-typeable Haemophilus influenzae (the patient had completed routine Hib vaccination) and Candida albicans was also present.

#### Recurrent pneumothorax

**Figure 3:**
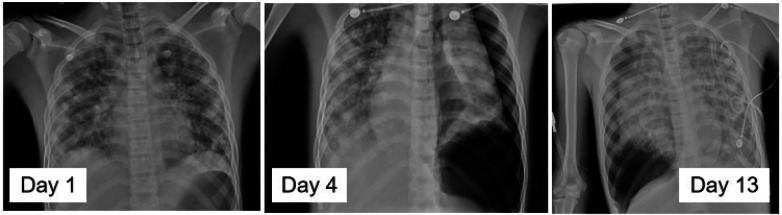
Radiographic findings of the patient's pneumothorax.

The patient experienced bilateral pneumothorax on days 4, 13, 20, and 42, each requiring closed thoracic drainage (Figure 3). Dynamic pathogenic monitoring revealed evolution of pulmonary pathogens from initial Staphylococcus aureus and H. influenzae to carbapenem-resistant Pseudomonas aeruginosa (CRPA).

#### Deep vein thrombosis

Vascular ultrasound on day 3 detected left lower extremity DVT, with subsequent progression, necessitating full-course anticoagulation.

### Diagnosis and multidisciplinary management

#### Dynamic anti-infective therapy

Based on serial cultures and metagenomic sequencing, the anti-infective regimen was adjusted over ten times, including vancomycin, linezolid, imipenem, tigecycline, piperacillin-tazobactam, amphotericin B, micafungin, and rifampin, following de-escalation principles.

#### Life support and organ protection

Prolonged mechanical ventilation, CRRT, multiple plasma exchanges, and blood transfusions supported organ recovery.

#### Multidisciplinary management

Orthopedics managed joint abscesses with ultrasound-guided aspirations and applied external plaster casts for pathological fractures; the patient was deemed unsuitable for extensive surgical debridement. Vascular surgery guided anticoagulation. Burn and plastic surgery directed wound care using moist healing principles with regular debridement and topical antibiotics. The patient's clinical course and major interventions are summarized in Table 1.

**Table 1:** Timeline of patient's clinical progression.

Date	Key Clinical Changes	Core Medical Interventions
2024-07-28	Admitted with septic shock, severe pneumonia, respiratory failure	Emergency intubation, mechanical ventilation, norepinephrine, empirical antibiotics (imipenem, vancomycin, micafungin)
2024-07-30	Ecchymosis and skin necrosis on left foot; DVT on ultrasound	Vascular surgery consultation; therapeutic anticoagulation initiated
2024-07-31	First left-sided pneumothorax	Closed thoracic drainage
2024-08-01	Bloody airway secretions	Bronchoscopy and bronchoalveolar lavage
2024-08-04	Persistent infection, fungal involvement per sequencing	Switched micafungin to amphotericin B
2024-08-15	CT: subcutaneous emphysema, pulmonary bullae; thrombus progression	Increased anticoagulation
2024-08-23	Necrosis and suppuration of left foot/calf	Burn surgery consultation; wound care with povidone-iodine, mupirocin, growth factor
2024-08-26	Left knee swelling, abscess on ultrasound	Ultrasound-guided aspiration (60 ml pus); confirmed cellulitis/septic arthritis
2024-09-20	Chest CT: multiple osteolytic lesions, sequestra, pathological fractures	Orthopedic consultation; conservative management (immobilization, antibiotics)
2024-09-29	x-ray: pathological fracture of right femoral neck	Long-leg plaster cast applied
Throughout	Recurrent fever, persistently positive pathogens (MRSA, *P. aeruginosa*, etc.)	Dynamic anti-infective adjustments (de-escalation and targeted therapy)

### Outcome

After more than 90 days of intensive treatment, the patient's condition stabilized. He was weaned off mechanical ventilation, organ function improved significantly, and infection was controlled. He was transferred to a general ward for continued rehabilitation. Long-term orthopedic follow-up is required.

## Discussion

### Limb necrosis as a sentinel sign of deep infection

In this case, limb necrosis was not an isolated endpoint but preceded septic arthritis and multifocal osteomyelitis. This clinical sequence suggests that necrotic tissue can become a persistent source of hematogenous dissemination. When limb necrosis develops in a child with septic shock, systematic screening of adjacent joints, bones, and deep soft tissues should be initiated promptly. Bedside ultrasound is an ideal initial modality for detecting effusions and abscesses in unstable patients (6, 7).

### Diagnostic and management implications

The multifocal osteomyelitis in this case represented hematogenous seeding during the septicemic phase, not contiguous spread. Diagnosis was not confirmed until day 54, by which time extensive osteolytic destruction and pathological fracture had already occurred. This delay underscores a practical lesson: in children with septic shock who develop persistent fever, localized pain, or swelling despite adequate antibiotics—particularly in the presence of a distal necrotic focus—clinicians should maintain a low threshold for advanced imaging (CT or MRI) when hemodynamic stability permits (8).

### Comparison with previously reported cases

Table 2 summarizes selected pediatric cases of sepsis-associated osteomyelitis and/or limb necrosis. As shown, the combination of limb necrosis progressing to multifocal osteomyelitis with pathological fracture is rarely documented, highlighting the unique progression in the present case.

**Table 2:** Comparison with previously reported pediatric cases of sepsis-associated osteomyelitis and/or limb necrosis.

Study	Age	Primary presentation	Limb necrosis	Osteomyelitis	Pathogen	Key intervention	Outcome
Treharne et al. (2003) (13)	2 years	Meningococcal septicaemia	Yes (distal limb)	Bone necrosis proximal to soft tissue	*N. meningitidis*	Bone scan-guided assessment	Below-knee amputation
Palácios et al. (2003) (14)	Various (*n* = 10)	Infectious diseases with DIC	Yes (multiple)	Bone necrosis reported	Mixed	Shock correction+thrombolytics	Variable, with handicaps
Khurtsilava et al. (2025) (15)	13 years	Hematogenous osteomyelitis	No	Yes (with DVT, septic PE)	Not isolated (suspected *Staphylococcus*)	Surgical intervention	Recovered
Hardgrib et al. (16)	13 years	MRSA sepsis	No	Yes (with arthritis, pneumonia)	MRSA	Antimicrobial therapy+repeated surgical debridement	Improved
Present case	12 years	Septic shock	Yes (left foot/calf)	Multifocal with pathological fracture	MRSA+H. influenzae+C. albicans	Ultrasound-guided drainage+conservative orthopedics	Stabilized after >90 days

### Multidisciplinary collaboration as a practical necessity

The successful stabilization in this case was achieved through sustained multidisciplinary collaboration involving intensive care, infectious diseases, orthopedics, vascular surgery, and wound care specialists. For clinicians managing similar cases, establishing a formal multidisciplinary team early—rather than consulting reactively as complications arise—is essential. This approach enables timely orthopedic evaluation before irreversible skeletal damage and facilitates real-time antibiotic adjustments based on evolving pathogen data (9–11).

### Role of point-of-care ultrasound

This case highlights the value of point-of-care ultrasound (POCUS) in critically ill children too unstable for transport to CT or MRI. Serial POCUS enabled early detection of DVT, joint effusions, and soft tissue abscesses, and facilitated safe ultrasound-guided drainage (8, 12). When septic arthritis or osteomyelitis is suspected in an unstable patient, POCUS serves as both a diagnostic and therapeutic guide.

## Conclusion

This case demonstrates that limb necrosis in pediatric septic shock may precede disseminated osteomyelitis. Early bedside ultrasound evaluation and multidisciplinary source control are critical when deep infection is suspected. Findings should be interpreted as hypothesis-generating given the limitations of a single case report.

## Data Availability

The original contributions presented in the study are included in the article/Supplementary Material, further inquiries can be directed to the corresponding author/s.
